# Exploring workability in an older working population: associations with cognitive functioning, sleep quality, and technostress

**DOI:** 10.3389/fpubh.2024.1303907

**Published:** 2024-04-29

**Authors:** Alice Fattori, Anna Comotti, Teresa Barnini, Cristina Di Tecco, Marco Laurino, Pasquale Bufano, Catalina Ciocan, Daniele Serra, Luca Ferrari, Matteo Bonzini

**Affiliations:** ^1^Occupational Medicine Unit, Foundation IRCCS Ca’ Granda Ospedale Maggiore Policlinico, Milan, Italy; ^2^Department of Occupational and Environmental Medicine, Epidemiology and Hygiene, Italian Workers' Compensation Authority (INAIL), Rome, Italy; ^3^Institute of Clinical Physiology, National Research Council, Pisa, Italy; ^4^Department of Public Health and Pediatrics, University of Turin, Turin, Italy; ^5^Department of Clinical Science and Community Health, University of Milan, Milan, Italy

**Keywords:** work ability index, successful aging, sleep disturbances, occupational health, shift work

## Abstract

**Objective:**

This observational study investigates workability and its associations with cognitive functioning, sleep quality and technostress among an older working population, also shedding light on potential differences between two occupational categories with different work schedules.

**Methods:**

Workers aged over 50, employed in different working sectors (banking/finance, chemical and metal-mechanic industry) were administered a self- report questionnaire including Work Ability Index (WAI), cognitive tests (Stroop Color Task, Corsi Blocks, Digit Span), sleep quality questionnaires (Pittsburgh Sleep Quality Index-PSQI; Insomnia Severity Index-ISI; Ford Insomnia Response to Stress Test-FIRST) and technostress scale. Linear regression models evaluated associations among variables, interaction effects investigated potential moderators.

**Results:**

A total of 468 aged workers categorized as white (WCWs; *N* = 289, 62%) or blue collars (BCWs; *N* = 179, 38%) were enrolled; most BCWs (*N* = 104; 58%) were night shift workers. WCWs reported higher workability, cognitive functioning, sleep quality and lower technostress (except for invasion and privacy subscales) than BCWs. Associations between cognitive functioning and workability were statistically significant only for BCWs [slopes equal to 0.2 (0.33), 0.8 (0.34), −0.02 (0.001) for Memory Span Corsi, Block Span Digit and Interference Speed respectively]; additionally, sleep quality significantly moderated this association (*p* = 0.007). Higher levels of technostress were associated with lower workability, and this relationship was stronger for BCWs.

**Conclusion:**

The aging of the workforce has important implications for occupational health and safety. Our findings suggest potential interventions and protective measures to promote older workers’ wellbeing; blue-collar workers particularly should benefit from tailored intervention to sustain workability and prevent technostress, considering the role of healthy sleep habits promotion.

## Introduction

The proportion of older workers in the workforce continues to increase globally, as individuals are living longer and are induced to work until older ages (1). Italy in particular is one of the oldest countries, with the largest gap between young and old workers among European countries: almost 21% of the population has exceeded 65 years of age and employment rates are 42.7% and 52.2% for younger and older workers, respectively (2). The aging of the workforce has important implications for both public and occupational health as older age may undermine physical ability, adaptability and general work effectiveness, also increasing the risk of occupational injuries (3). These trends highlight the importance to investigate how work affects, and is affected by, health and well-being of older adults.

Successful aging at work has attracting growing research interest as it aims to maintain older workers’ health, motivation and workability and to promote adaptive recovery from decline (4). Workability was first conceptualized by the Finnish Institute of Occupational Health as the perceived likelihood of completing habitual work tasks in accordance with work demands, state of health, physical and mental abilities (5). It is a multidimensional construct that can be described as a complex interaction between individual determinants such as health, attitude, mental and physical abilities and work environment; workability can be influenced by several variables including physiological changes, individual and contextual moderators and it is associated to a variety of work outcomes (such as turnover, job performance, occupational well-being) as it is considered to form the basis of productivity and well-being at work (6, 7).

In this framework, cognitive functioning has been identified as an important predictor of workability (8). Cognitive functioning is related to job performance because of its influence on workers’ capacity to learn new knowledge and skills necessary to carry out work-related functions and, in turn, to remain productive in work environments that require continuous skill learning (9). Aging cognitive changes are mainly related to a general and progressive decline that primarily impact some crucial cognitive functions such as the rate of perceptual speed, the working memory, the inhibitory functions, and the sensory functions (10) and that therefore may lead to important differences in job performance between younger and older adults (8).

In addition to cognitive functioning, workability can also be affected by perceived physical and mental health as well as by different work-related psychosocial risk factors, such as job demands and work-life balance (7, 11). All these factors could lead to important differences among occupations, particularly considering shift workers. Shift workers report lower work-life balance satisfaction than day workers, as working atypical hours can limit social life, contribute to work-life conflict, and affect psychological well-being (12). Moreover, shift work, especially nightshift, makes difficult to maintain a typical sleep schedule, disrupting sleep duration, timing, and resulting in the alteration of circadian rhythms and sleep homeostasis, with a long-term effect on the risk for several chronic health conditions typically occurring in older age (13, 14).

Insomnia is one of the most common health problems among the working population, as 30–50% occasionally experience insomnia symptoms and up to 10% meet the criteria for a clinical diagnosis of insomnia (15). A poor sleep quality may negatively influence one’s workability as it affects the quality of life, the cognitive functioning, and the productivity (16). Many studies have established that sleep disorders can lead to reduced physical and mental functioning, including fatigue, anxiety, difficulties in performing complex tasks, reduced job satisfaction and work performance (17).

Additionally, as the workplaces become more digitized and reliant on Information and Communication Technologies (ICT), workers may experience higher levels of technostress (18). Increased levels of technostress can have impact with adverse health and work outcomes, and significantly worsen workers’ attitudes toward work, leading to decreased job satisfaction and organizational commitment (19). Older workers particularly might face potential challenges in adapting to and utilizing technology in the workplace and recent studies suggested to consider technostress a potential risk factor for the well-being of older workers (20). However, the adverse effects of technology on the workability of older workers have received insufficient investigation, necessitating further studies to explore the underlying causes and consequences in order to identify potential interventions.

All in all, the literature on workability among older workers is still limited and we deemed it worthwhile to investigate its interplay with cognitive functioning, sleep quality and technostress as all these factors pose significant challenges to the health and safety of older workers. Therefore, the aim of this study is to investigate workability through a multidimensional approach specifically focusing on its associations with cognitive functioning, sleep quality, and technostress in an aging workers population. In addition, by considering different occupational setting, this study also aims to shed light on the potential differences in workability challenges faced by individuals in different occupational categories.

## Methods

We designed an observational study including workers aged over 50. The study was conducted in workplaces where the Occupational Medicine Unit of IRCCS Ca′ Granda Ospedale Maggiore Policlinico Foundation and the Occupational Medicine Unit of University of Turin carried out the medical surveillance required by Italian Legislation in terms of occupational safety (i.e., Legislative Decree n.81/2008); we enrolled subjects employed in banking and finance sector, chemical and metal-mechanic industry to include different work environments. The detailed study protocol and the full description of questionnaires and variables were published elsewhere (21).

During the medical surveillance required by the current Italian Legislation (Legislative Decree 81/08 and s.m.i.), the occupational physician invited eligible workers to participate to the study. Inclusion criteria were age over 50, full-time schedule and a job seniority of at least 10 years in their current workplace; there were no exclusion criteria based on gender, ethnicity, or clinical characteristic. Subjects who signed the written consent were enrolled and identified with a pseudo-anonymous code. The occupational physician conducted in-person interviews collecting workers’ socio-demographic (age, gender, weight, height, pack/years) and occupational information (occupational role, job seniority, shift work, remote work, work-related history, exposure to risk factors) through the REDCap platform (22). The worker was administered a self- report questionnaire including the Work Ability Index (WAI) (5) and a technostress measure specifically developed for older adults (20). The WAI questionnaire takes into account psychosocial and physical factors related to work, employee’s mental and physical resources and his/her health condition as it investigates seven dimensions: (1) the individual’s current work ability compared with their lifetime best; (2) work ability in relation to the demands of the job; (3) number of diagnosed illnesses; (4) estimated impairment due to diseases/illnesses or limiting conditions; (5) amount of sick leave during the past 12 months; (6) personal prognosis of work ability 2 years prior the study; (7) estimate of mental resources. A total score (i.e., Index) is calculated by summing up the scores of all items, ranging from 7 to 49; the Index categorizes work ability as poor (7 to 27 scores), moderate (28 to 36 scores), good (37 to 43 scores) and excellent (44 to 49 scores).

The Technostress measure was specifically developed to examine the stress induced by Information and Communication Technology (ICT) use among older adults (20). For this study, we adapted original items in order to measure technostress caused by technologies used at and for work. It comprises 14 items that pertain to 5 dimensions: overload (facing an excess of challenges and, consequently, executing tasks more slowly), invasion (intrusion of ICT into everyday life due to blurred boundaries between work and private life), complexity (experiencing difficulties to cope with complexity and ongoing changes ICT), privacy (threat to personal information, risk of data monitoring and tracing), and inclusion (perceiving a sense of inferiority compared to younger ICT users). Participants respond using a five-point Likert scale, ranging from 1 (strongly in disagreement) to 5 (strongly in agreement). Five scores were computed for each dimension by averaging the corresponding item scores, and a total technostress score was calculated by summing up the five sub-scores.

Once completed these questionnaires, participants underwent a cognitive assessment through three cognitive tests: Stroop Color Task (23), Corsi Blocks test (24) and Digit Span (25) implemented on a mobile tablet application, measuring their ability to inhibit semantic cognitive interference, visuo-spatial and verbal short-term memory respectively; higher scores suggest worse (Stroop Color Task) or better (Corsi Blocks test, Digit Span) cognitive functioning. Questionnaires on sleep quality and habits were later completed by subjects on their own; we adopted the Pittsburgh Sleep Quality Index-PSQI (26), the Insomnia Severity Index-ISI (27) and the Ford Insomnia Response to Stress Test-FIRST (28). For each questionnaire a global score was calculated by summing different component scores; higher scores indicate worse sleep quality.

### Statistical analysis

All answers were collected into a merged database, preliminary synthesized through frequencies and percentage or mean and standard deviation. Subjects employed in the chemical/metal-mechanic industry mostly performing physical/manual labor or in the banking/finance sector performing managerial or administrative work were categorized as blue collars workers (BCWs) or white collars workers (WCWs) respectively. Possible differences in questionnaires and test results between groups were tested by Student *t*-test. Linear associations between variables were assessed through linear regression models, adjusted by gender, education level and shift work. Interaction effects of regression models investigated potential continuous (sleep habits) or categorical (type of job) moderators.

The R software was used for the analyses. A *p*-value <0.05 was considered as significant.

## Results

A total of 468 aged workers participated and completed the questionnaire between November 2021 and January 2023. Participation rate was 80.5 and 79.6% for, respectively, WCWs and BCWs, and similar across age. Sample characteristics are presented in Table 1. Participants were predominately males (*N* = 354; 76%) with median age of 55 years (IQR 52–58). Half of the workers (*N* = 231; 49%) were bank employees; 134 (29%) worked in a chemical industry and 103 (22%) in a mechanical industry; subjects were categorized as white (WCWs; *N* = 289, 62%) or blue collars (BCWs; *N* = 179, 38%) according to their occupational sector. More than half of WCWs (54.5%) were graduates or post-graduates, while BCWs were similarly divided between secondary school (48.5%) and 8th grade (46.5%) diploma. One hundred and four workers (22%) were night-shift workers and 68 (15%) had been night-shift workers before enrolment time; exposure to loud noise (37%), chemicals (35%) and heavy lifting (32%) were the most common workplace hazards reported by participants. More than half of all participants (244; 52%) reported to suffer from one or more diseases and the most frequent disease was arterial hypertension.

**Table 1:** Frequencies and percentages of sociodemographic and occupational characteristics of study sample and across subgroups (blue collars workers-BCWs and white collar workers-WCWs).

	Whole sample *N* (%)	BCWs *N* (%)	WCWs *N* (%)
TOT	468	179 (38)	289 (62)
Gender			
Male	356 (76)	171 (96)	185 (64)
Female	112 (24)	8 (4)	104 (36)
Education level			
8^th^ grade diploma	203 (43)	158 (54.5)	9 (5)
High school diploma	98 (21)	120 (41.5)	83 (46.5)
Academic degree	167 (36)	11 (4)	87 (48.5)
Job area			
Banking and finance	231 (49)	1 (1)	230 (80)
Industry	237 (51)	178 (99)	59 (20)
Night shift-work			
No	294 (63)	30 (17)	265 (92)
Past	68 (15)	45 (25)	24 (8)
Current	104 (22)	104 (58)	-
Smokers			
No	292 (62)	80 (45)	211 (73)
Past	106 (23)	62 (35)	44 (15)
Current	71 (15)	37 (20)	34 (12)
Occupational risk exposure (one or more)			
Noise	175 (37)	136 (76)	15 (5)
Heavy lifting	151 (32)	62 (35)	4 (1)
Chemical risk	166 (35)	148 (83)	18 (6)
High temperature	66 (14)	62 (35)	4 (1)
Other	104 (22)	49 (27)	55 (19)
Remote working			
No	188 (40.5)	161 (90)	26 (9)
Past	25 (5.5)	10 (6)	16 (6)
Current	253 (54)	8 (4)	246 (85)

Table 2 shows the summary statistics of workability, technostress, cognitive functioning and sleep quality in the total sample and by occupational groups. The mean WAI score was 42.8 (sd = 4.9), which corresponds to a good level of workability, with no statistical differences according to participants’ gender. Mean WAI score also differed considering education level (8th grade diploma = 40.2, high school diploma = 42.7, academic degree = 44.8, *p* < 0.001). When categorized based on their scores, 53% of workers reported an excellent workability, 39% a good workability and 8% moderate or low workability. Participants’ total technostress (Cronbach’alpha = 0.75) ranged from 7 to 20 (mean = 12.9, sd = 2.1), revealing different levels of stress among older technology users. In particular, results showed a higher mean in the inclusion subscale (mean = 3.3, sd = 0.6) compared to overload (mean = 2.3, sd = 0.7) and privacy (mean = 2.3, sd = 0.8) subscales. The cognitive functioning resulted preserved in each measured skill (Table 2). Regarding sleep habits, results showed poor sleep quality (PSQI >5), absence of insomnia (ISI <7) and sleep reactivity (FIRST >16), with means just above relevant cutoffs.

**Table 2:** Mean value, sd, and scale range of Work Ability Index, technostress, cognitive functioning, sleep quality in the whole sample and across blue collars (BCWs) and vs. white collars (WCWs) workers sub-samples (*t*-test: *p < 0.05, **p < 0.01, ***p < 0.001).

	Whole sample		BCWs	WCWs
	Mean (sd)	Range	Mean (sd)	Mean (sd)
Work Ability Index (WAI)***	42.8 (4.9)	15–49	40.1 (5.8)	44.4 (3.1)
Current workability compared with the lifetime best***	8.2 (1.4)	1–10	7.8 (1.6)	8.4 (1.1)
Workability in relation to the demands of the job***	8.9 (1.3)	2–10	8.3 (1.6)	9.3 (1.0)
Current disease diagnosed by a physician***	5.2 (1.5)	1–7	4.6 (1.6)	5.5 (1.4)
Estimated work impairment due to disease***	5.8 (0.6)	2–6	5.6 (0.8)	5.9 (0.5)
Sick leave during the past year (12 months) ***	4.3 (0.9)	1–5	4.0 (1.1)	4.5 (0.8)
Own prognosis of workability two years from now***	6.7 (1.1)	1–7	6.4 (1.6)	6.9 (0.5)
Mental resources***	3.6 (0.6)	1–4	3.4 (0.7)	3.8 (0.5)
Technostress*	12.9 (2.1)	7–20	13.1 (2.1)	12.7 (2.0)
Overload***	2.3 (0.7)	1–5	2.6 (0.8)	2.1 (0.6)
Invasion***	2.5 (0.8)	1–5	2.3 (0.8)	2.7 (0.8)
Complexity**	2.4 (0.7)	1–5	2.6 (0.7)	2.4 (0.6)
Privacy*	2.3 (0.8)	1–5	2.2 (0.6)	2.3 (0.7)
Inclusion**	3.3 (0.6)	1–5	3.4 (0.7)	3.2 (0.6)
Cognitive functioning				
Memory Span Corsi**	4.9 (1.1)	1.5–7.5	4.6 (1.2)	5.0 (1.1)
Block Span Digit***	6.1 (1.3)	3–8	5.6 (1.3)	6.4 (1.3)
Interference speed	−2.2 (98)	−624–296	−5.0 (99.2)	−0.6 (98.2)
Sleep quality				
Sleep quality (PSQI)*	5.8 (3.3)	0–18	6.3 (3.3)	5.5 (3.3)
Insomnia (ISI)**	6.3 (5.1)	0–23	7.5 (5.4)	5.7 (4.8)
Sleep Reactivity (FIRST)	16.6 (4.7)	9–36	16.6 (4.9)	16.6 (4.7)

WCWs and BCWs showed statistically significant mean differences in each variable, with WCWs reporting higher workability, lower technostress (except for invasion and privacy subscales), higher cognitive functioning, better sleep quality and lower insomnia than BCWs.

**Figure 1:**
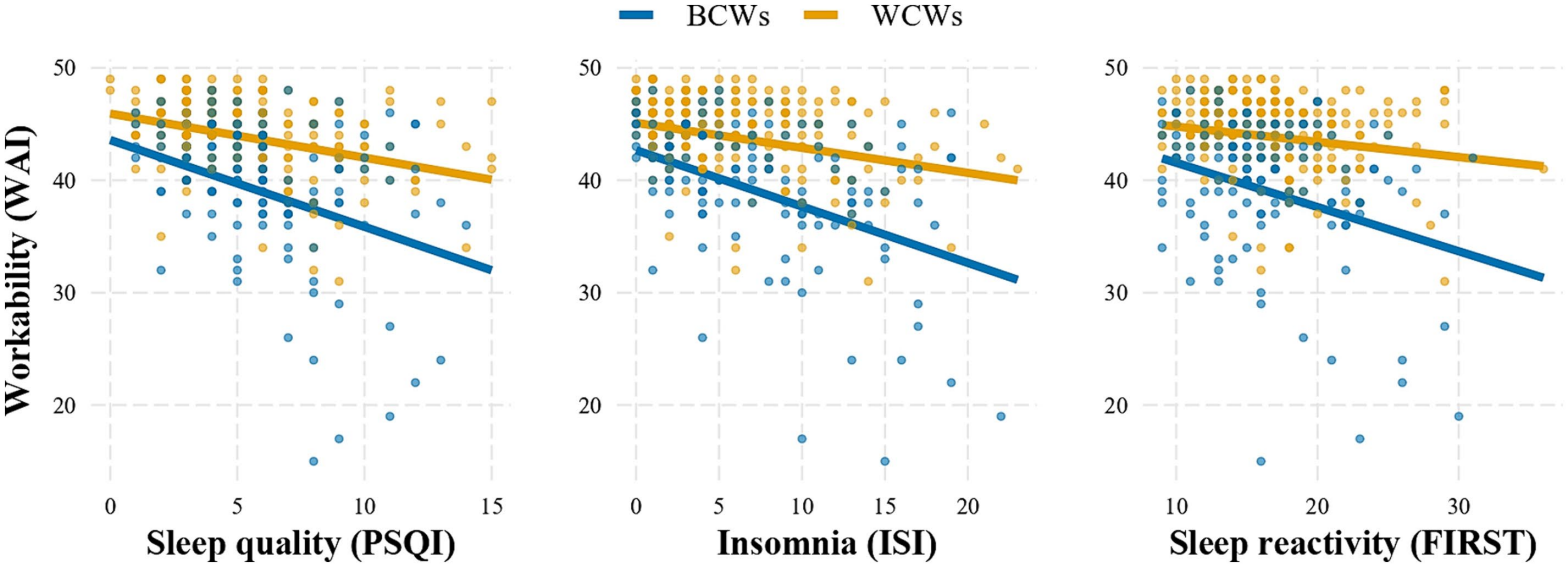
Adjusted linear regression models of sleeps quality (measured by PSQI, ISI, FIRST) on workability (WAI) moderated (*p* < 0.001) by type of job (blue collars; BCWs vs. white collars; WCWs).

The association between workability and sleep habits is depicted in Figure 1. Based on a simple linear regression model, we found sleep quality, insomnia and sleep reactivity scores to be negatively and significantly associated with workability (*b* = −0.6, *p* < 0.001, *b* = −0.4, *p* < 0.001, and *b* = −0.3, *p* < 0.001 respectively). A significant interaction was found between sleep habits and occupational groups in relation to workability after adding the interaction term, showing that the type of job moderated the association between sleep-related and WAI scores. In particular, such inverse relationship was significantly stronger for BCWs (with slopes equal to −0.6 (0.16), −0.5 (0.07), −0.4 (0.07) for sleep quality, insomnia and sleep reactivity respectively) than for WCWs (with slopes equal to −0.5 (0.11), −0.2 (0.05), −0.1 (0.07) for sleep quality, insomnia and sleep reactivity respectively).

**Figure 2:**
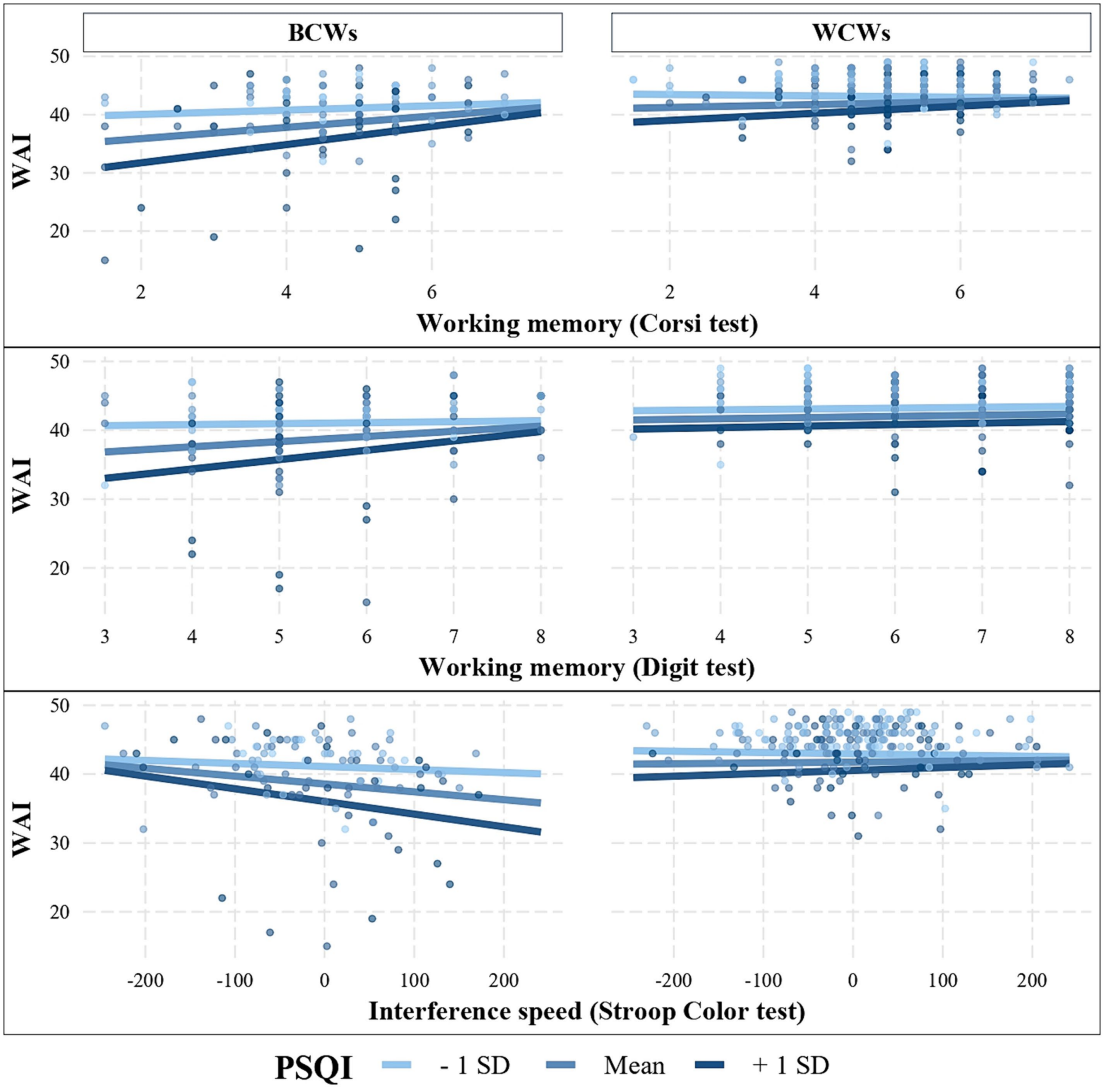
Adjusted linear regression models of cognitive functioning (Corsi test, Digit test, Stroop Color test), on workability (WAI) moderated by type of job (blue collars; BCWs vs. white collars; WCWs; *p* < 0.001) and by sleep quality (PSQI; mean, + sd, −sd; *p* = 0.007).

Figure 2 shows linear regression between cognitive tests and WAI scores; overall results showed an increase in WAI scores as memory test scores improved (*b* = 0.6, se = 0.2, *p* = 0.009, and *b* = 0.4, se = 0.2, *p* = 0.04 for Memory Span Corsi and Block Digit Span scores respectively) however the relationship with interference speed was not statistically significant (*b* = −0.001, non-significant). Based on slopes analyses, we found that the association between workability and cognitive functioning was statistically significant only for BCWs [slopes equal to 1.2 (0.33), 0.8 (0.34), −0.02 (0.001) for Memory Span Corsi, Block Span Digit and Interference Speed] as slopes were very close to zero among WCWs (with); furthermore, sleep quality significantly moderated the association between the WAI and cognitive scores (*p* = 0.007).

Higher levels of technostress were associated with lower workability (*b* = −0.6, se = 0.1; *p* < 0.001); this relationship was stronger for BCWs (slope equal to −0.6, se = 0.2) than for WCWs (slope equal to −0.3, se = 0.1), even if the difference was not significant.

## Discussion

In this observational study, we examined the associations among workability, cognitive functioning, sleep quality and technostress in a population of older workers (age ≥ 50 years). To the best of our knowledge, this is one of the first study focusing on the relationship of cognitive abilities, sleep quality and technostress on workability among older workers comparing subjects employed in different occupational sectors.

Overall, we found good average workability among participants, with subjects employed as white collar reporting higher scores compared to blue collars across all WAI subscales, thus suggesting better prognosis of workability for the future, higher mental resources, less sick leave for participants employed in the banking and finance sector; this result is consistent with the existing literature showing that blue collar employees are more likely than white collar to report lower workability (29, 30). It is worth noting that, in our sample, blue collars reported overall good workability regardless of age, with higher average score compared to a previous study conducted among Italian aged blue collars workers (31); these findings may be explained by taking into account that WAI scores can be considerably affected by personal health status, which resulted as optimal in this sample (32).

Cognitive abilities were also higher for WCWs compared to BCWs, particularly concerning the Digit Span task. According to literature, cognitive abilities among workers may be primarily influenced by education levels and occupational factors such as tasks’ complexity, which can presumably be greater in white collars, although some authors suggest taking into account also the positive impact of off-work activities as participation in social and intellectual activities (33). Several studies and theories showed that older adults could compensate this decline through cognitive strategies and problem-solving skills developed in stimulating and complex environments (e.g., cognitively demanding environments) (6, 34).

Results showed a positive association between all cognitive abilities investigated and WAI score in the total sample; however, when comparing subgroups, this association remained significant only for blue collars. During the last decades, many studies have examined the link between the cognitive complexity of works and workers’ level of cognitive functioning, showing that working in complex jobs, with a high level of mental job demands, is typically related to a better cognitive functioning in later life and with a lower prevalence of cognitive impairment and dementia (35). On the other hand, jobs with higher physical demands are usually associated with a greater cognitive decline and higher risk of dementia (36). A possible interpretation for this pattern is that physically demanding jobs, often characterized as blue collar works, are typically associated with the cognitive correlates of lower socioeconomic status, educational attainment, and income (37).

Additionally, the cognitive reserve hypothesis (34), a well-established theory in the field of neuroscience, suggests that our new knowledge and learning are associated with increased neuronal development. Indeed, each mentally stimulating experience occurred in life (e.g., cognitive engagement, education, social stimulation) helps building additional neuronal resources and cognitive strategies, the so-called cognitive reserve, which in turn may increase individual’s resilience against neuronal loss and cognitive decline. White collars could therefore have a greater cognitive reserve, which maintains higher cognitive functioning compared to blue-collars and prevents the cognitive decline associated with aging. All these factors are consistent with Ilmarinen’s theory according to which workability is determined by the specific work demands and the perceived personal resources available to meet them (5).

Sleep quality also had a positive association with WAI scores and, similarly to cognitive abilities, this association was stronger among blue collars. Interestingly, we found that impairment in sleep quality increased the association between cognitive abilities and workability, supporting the moderating role of sleep habits; this finding may pose a challenge to blue collars workers as we found poorer sleep quality among them compared to white-collar workers. Furthermore, literature suggests that blue collar workers could experience a decreased sleep quality compared with individuals in other occupations and this association may be due to higher exposure to loud noise and physical demands at work, lower socioeconomic income and limited access to healthcare resources, higher BMI (38–41). Additionally, night shifts can affect sleep quality by disrupting the circadian rhythm, leading to sleep deprivation as well as impaired health and performance (42).

These results encourage workplace interventions to sustain sleep quality among older employees, particularly among blue collars workers. Workplace health promotion (WHP) intervention are usually aimed at maintaining and promoting health and work ability through organizational and training programs to improve lifestyle and health education and they have also been shown to be effective in favorably affecting sleep habits (43). Although workplace is largely recognized as an ideal setting for implementing health promotion due to the time spent by individuals at work, social support networks offered by colleagues and existing communication formats, a special attention should be paid to tailor intervention toward older workers, as literature suggest they may be less likely to participate in WHP (44): in this regard, we recommend adopting a multimodal approach to change, providing ongoing support for workers’ participation, offering clear explanations of the effectiveness of health promotion initiatives, and adopting an integrated approach that combines occupational risks prevention measures (45).

In this study, the average total score on technostress questionnaire measure resulted similar to those found by Nimrod (20) among subjects aged 60 years and over; however, in our sample, blue collars reported slightly higher level of technostress compared to white collars. Particularly, the technostress subscales analysis highlighted that WCWs reported higher scores in invasion and privacy sub-scales while BCWs experienced greater stress due to work-overload, excessive complexity of technologies and a perceived greater inability in understanding technological tools. These findings are consistent with the different types of technologies mainly used in each occupational sector. WCWs are usually engaged in more administrative tasks, supported by the use of computers and informatics system, which can easily exceeds the boundaries of work thus invading their private life. Differently, BCWs may face challenges to cope with ITC only within their working time however with higher strain as they have been predominantly interacting with them only in recent years as a consequence of the fourth industrial revolution (Industry 4.0), which is including ICT in automation and real-time data exchange (e.g., semi-automatic production, computer numerical controls). Additionally, technostress was found to be inversely associated with WAI scores indicating that higher levels of technostress were associated with lower workability; this association was slightly stronger for blue collars.

Studies exploring technostress among older workers are limited, which makes it challenging to compare the current findings with previous research; however, scholars have pointed out the detrimental impact of technostress on life satisfaction and well-being suggesting that individuals in later life may have fewer resources to cope with ICT stress compared to those who are more proficient with technology as “native-digital” (20). Better insights into techno-stressors, as well as interventions to effectively manage them, are strongly encouraged.

This study has some limitations. Although selection criteria aimed at enrolling subjects aged over 50, final sample was homogenous according to age, as most participants were from 52 to 57 years old thus making comparison between age ranges (e.g., with workers aged over 60) difficult; additionally, as reported by WAI scores, health status among participants was generally good, with few diagnosed diseases, restricting results’ generalizability to a healthy population. Our findings suggested associations among cognitive abilities, sleep quality, technostress and workability without determination of causality, future research according to the study protocol would explore longitudinal associations. Additionally, sleep quality data relied on participants’ perceptions without reference to objective measures (e.g., actigraphy). We are aware that subjective and self-report measures may entail for common method-bias and may be associated to unreliable answers; in order to minimize these risks, dedicated occupational physicians with prolonged experiences as company doctors conducted interviews and collected participants’ answers. Additionally, cognitive performance was assessed through objective measures to overcome potential bias related to perceived cognitive impairment. In this respect, this is one of the first study aimed at evaluating workability among older worker with a multidimensional approach, adopting both subjective and objective measures, with data collected on site with specialized health care professionals, and with a very high participation rate.

## Conclusion

Social and technological transformations pose a considerable challenge to workers’ health and safety, especially for aged workers. Our findings contribute to improve the understanding of interactions among workability, sleep disturbances, cognitive performance and technostress in two different work settings and professionals. These results also suggest potential interventions and protective measures to promote the well-being of aged workers. Blue-collar workers should benefit from tailored intervention to sustain workability and prevent technostress, considering the role of healthy sleep habits promotion.

## Data availability statement

The raw data supporting the conclusions of this article will be made available by the authors, without undue reservation.

## Ethics statement

The study was approved by the Ethical Committee of the Foundation IRCCS Ca′ Granda Ospedale Maggiore Policlinico on June 22, 2021 (Milan Area 2 Ethical Committee, with decree number 616_2021bis). The studies were conducted in accordance with the local legislation and institutional requirements. The participants provided their written informed consent to participate in this study.

## Author contributions

AF: Conceptualization, Methodology, Writing – original draft, Writing – review & editing. AC: Conceptualization, Data curation, Formal analysis, Methodology, Writing – original draft, Writing – review & editing. TB: Writing – original draft, Writing – review & editing. CT: Conceptualization, Funding acquisition, Methodology, Writing – review & editing. ML: Conceptualization, Methodology, Software, Writing – review & editing. PB: Data curation, Formal analysis, Methodology, Software, Writing – review & editing. CC: Conceptualization, Investigation, Writing – review & editing. DS: Writing – review & editing. LF: Conceptualization, Writing – review & editing. MB: Conceptualization, Methodology, Project administration, Supervision, Writing – original draft, Writing – review & editing.
